# Root exudate chemical cues of an invasive plant modulate oviposition behavior and survivorship of a malaria mosquito vector

**DOI:** 10.1038/s41598-021-94043-5

**Published:** 2021-07-20

**Authors:** Trizah K. Milugo, David P. Tchouassi, Reginald A. Kavishe, Rhoel R. Dinglasan, Baldwyn Torto

**Affiliations:** 1 International Centre of Insect Physiology and Ecology (icipe), P.O Box 30772-00100, Nairobi, Kenya; 2 Kilimanjaro Christian Medical University College (KCMUCo), P.O Box 2240, Moshi, Tanzania; 3 Department of Infectious Diseases & Immunology, College of Veterinary Medicine, Emerging Pathogens Institute, University of Florida, 2055 Mowry Road, Gainesville, FL USA

**Keywords:** Chemical biology, Ecology, Chemistry

## Abstract

Gravid female *Anopheles gambiae* mosquitoes identify suitable oviposition sites through a repertoire of cues, but the influence of allelochemicals, especially root phytochemicals in modulating this behavior and impacting subsequent progeny bionomics remains unexplored. We addressed these questions in the malaria vector *Anopheles gambiae* and its invasive host plant *Parthenium hysterophorus*. Using chemical analysis combined with laboratory behavioral assays, we demonstrate that a blend of terpenes, namely α-pinene, α-phellandrene, β-phellandrene, 3-carene and (*E*)-caryophyllene emitted from *P. hysterophorus* root exudate treated-water attracted gravid females. However, fewer eggs (55%) hatched in this treatment than in control water (66%). The sesquiterpene lactone parthenin, identified in both the natural aquatic habitat harboring *P. hysterophorus* and root exudate-treated water was found to be responsible for the ovicidal effect. Moreover, larvae exposed to parthenin developed 2 to 3 days earlier but survived 4 to 5 days longer as adults (median larval survival time = 9 days (all replicates);11 to 12 days as adults) than the non-exposed control (median larval survival time = 11 days (reps 1 & 2), 12 days (rep 3); 6 to 7 days as adults). These results improve our understanding of the risk and benefits of oviposition site selection by gravid *An. gambiae* females and the role root exudate allelochemicals could play on anopheline bionomics, with potential implications in malaria transmission.

## Introduction

The mosquito *Anopheles gambiae* is considered one of the most efficient vectors of malaria parasites in sub-Saharan Africa^1^. According to the World Health Organization (WHO), there were an estimated 229 million cases of malaria worldwide, with over 400,000 deaths in 2019^2^. Additionally, the WHO reported that the burden of the disease was disproportionately higher in Africa, with 94% of the reported cases and deaths^2^. Although malaria control is comprised of a combination of approaches including vector control, surveillance, early disease diagnosis and treatment, vector control remains the most cost-effective disease intervention^3^. Vector control mainly involves use of topical or oral insecticides to target host-seeking mosquitoes. However, recent research has focused on the development of odor baits to improve outdoor control of mosquitoes^4^—an approach which requires identification of key olfactory cues that mediate mosquito host and nectar-seeking and oviposition behavior.

Oviposition site selection is a common evolutionary trait in insects including malaria vectors, and is critical for their fitness and survival. Gravid females of the malaria vector may oviposit in aquatic habitats containing immature conspecifics, high food availability (e.g. microbes)^5,6^, fewer predators (e.g. backswimmers) and fewer competitors (e.g. tadpoles)^5^. Additionally, gravid females may assess physicochemical characteristics of the aquatic habitat (e.g. color, temperature, optical density, chemistry) before laying their eggs^6^. For oviposition site chemistry, recent studies have identified microbe-derived volatile organic compounds (VOCs) such as cedrol from the fungus *Fusarium *sp.^7,8^ as attractants for gravid *An. gambiae* s.s. The VOCs α-pinene, nonanal, limonene and benzaldehyde identified from rice and sugarcane pollen have also been found to attract gravid females of *An. arabiensis*^9,10^. These biotic and abiotic factors are all significant because they can impact adult mosquito life history traits, survival and vectorial capacity^11,12^.

In addition to volatile semiochemicals, non-volatile chemicals, sprayed on vegetation growing near mosquito breeding sites may play a role in gravid female oviposition response and juvenile survival^4^. Additionally, phytochemicals may be released into depressions or pools harboring juvenile stages of mosquitoes affecting larval density^13^. *Parthenium hysterophorus* is an invasive plant that grows in areas prone to flooding^14^. Indigenous to the Americas, this plant has spread widely in East Africa, and commonly noted in Kenya, it has invaded vast tracts of land in the western part of the country, a high endemic area for malaria^15^. It has also been identified as a preferred nectar source for energy by *An. gambiae*^16,17^. Although the volatiles that attract nectar-seeking adult females to the plant is known, comprised of a blend of hexanal, β-pinene, limonene, and linalool oxide^4,16^, how *P. hysterophorus* allelochemicals potentially influence oviposition site selection in *An. gambiae* remains to be explored. In this study, we investigated the influence of the plant’s root exudate chemicals on *An. gambiae* oviposition and bionomics to explore its potential application in malaria control.

## Methods

### Experiments using *P. hysterophorus* root exudate water samples

#### Insects for oviposition bioassay

*Anopheles gambiae* mosquitoes (Mbita strain) used in this study were from a colony established in 2018 and maintained at the International Centre of Insect Physiology and Ecology (*icipe*), Duduville Campus, Nairobi, Kenya. The adults were reared using standard insectary conditions at 28 ± 2 °C, and 72% relative humidity (RH) under a photoperiod of 12 h: 12 h (L:D). For egg development, the mosquitoes were offered human blood through arm feeding, one to two times weekly and had ad libitum access to a 6% glucose solution (wt:vol). The blood fed females were kept together with males for 2 days and used on the third day for the oviposition experiment. Eggs were laid in oviposition cups (7 cm top diameter, 4 cm base diameter and 7 cm depth) lined with filter paper (Whatman 90 mm, GE Healthcare UK Ltd, Buckinghamshire, London, UK) and transferred to larval rearing trays (39 × 28 × 4 cm depth). Upon hatching, the larvae were separated into groups of ~ 500 larvae per rearing tray and fed on commercial Tetramin^®^ fish food (Tetra, Germany). The rearing room was maintained at 32 ± 2 °C, and 52% RH during the day, and 24 ± 2 °C and 72% RH at night with a photoperiod of L:D 12:12 h^15^. The rearing water was changed after every 2 days. Pupae were transferred daily to emergence cups containing 15 mL water and placed in a new cage (15 × 15 × 15 cm). Emerged adults (1 day old) were maintained on 6% glucose solution as described above.

#### Collection of root exudate water samples from wild growing *P. hysterophorus*

Root exudate water samples were collected from wild growing *P. hysterophorus* (~ 30, 60 and 90 cm tall) on the *icipe* campus and used in various biological assays (i.e., oviposition response, mosquito growth and development assays). Briefly, wild growing *P. hysterophorus* were transplanted into a plastic pot (23 cm top diameter, 12 cm base diameter and 22 cm depth; batches of ten plants per pot in garden soil from the *icipe* campus) and watered with rainwater to obtain root exudate water. The root exudate water was collected from the plants after 1 week: to allow the plants to stabilize and to eliminate possible contamination of it with defense compounds released in response to uprooting. Water obtained from potted soil (same volume as used for the potted *P. hysterophorus*) without *P. hysterophorus* plants but from the same site, served as control water.

#### Oviposition response assay with root exudate water

**Figure 1:**
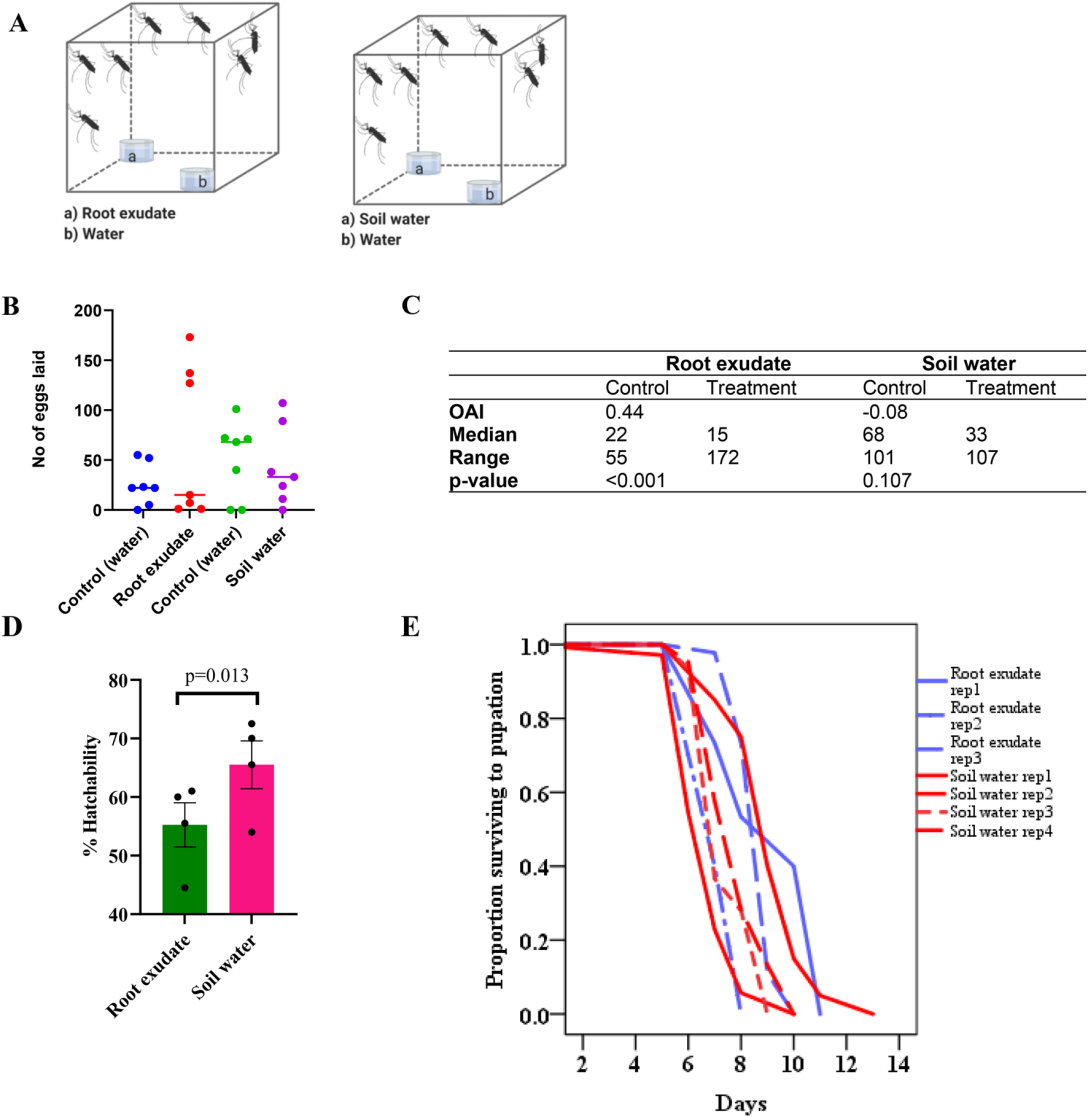
Effect of root exudate water on oviposition response and aquatic stage development of *An. gambiae.* (**A**) A schematic representation showing the setup of the oviposition experiment using root exudate water and soil water. (**B**) Oviposition activity of root exudate water and soil water. (**C**) Table summarizing median number of eggs laid and the range. (**D**) Egg hatch rates was low in root exudate water compared to soil water. (**E**) Time to pupation (days) for larvae exposed to root exudate water relative to soil water.

Root exudate water was evaluated for its ability to influence oviposition response of gravid females in a dual-choice assay (Fig. 1A). The gravid females (n = 12 as described in Dieter et al*.*^18^; electronic supplementary material, methods) were presented a choice between the treatment and distilled water contained in similar polypropylene cups and the number of eggs laid counted using a microscope (Leica M127, Switzerland) after every 24 h for four consecutive days^7^. The cups were placed in opposite corners of the experimental cages and their positions interchanged every 24 h to avoid positional bias. Each cup was lined with a filter paper (Whatman 90 mm, GE Healthcare UK Ltd, Buckinghamshire, London, UK) and filled with 30 mL of the test solution (to keep the filter paper moist; as previously described in^19^) or an equivalent volume of distilled water as the control. A similar experiment was performed using water obtained from soil without *P. hysterophorus* plants (Fig. 1A). The bioassays were performed in triplicate as previously described in Ilahi et al*.*^20^ and repeated once.

#### Headspace collection and analysis of root exudate volatiles

To collect headspace volatiles, 500 mL of the root exudate water were placed in air-tight glass jars and activated charcoal-filtered and humidified air passed over it. The volatiles were collected for 24 h on three pre-cleaned (dichloromethane (DCM) and nitrogen-dried) Super Q adsorbent filters (30 mg each, Analytical Research System, Gainesville, Florida, USA) at a flow rate of 170 mL/min. The three Super-Q filters (each treatment), were each eluted with 200 µL GC-grade DCM (Sigma Aldrich, St. Louis, Missouri, USA) into 2 mL clear glass vials, each containing 250 µL conical point glass inserts (Supelco, Bellefonte, PA, USA) and immediately analyzed by coupled gas chromatography/mass spectrometry (GC/MS). Also, natural water from depressions harboring growing *P. hysterophorus* in the field was sampled during the rainy season (described under parthenin detection) to allow comparison of its volatiles with that of the root exudate water. Volatiles collected from distilled water served as negative controls. Compounds were identified by comparison of their mass spectral data with library data: Adams2, Chemeco and NIST11 and confirmed with those of authentic samples. The relative peak area (%) of each constituent as generated by the NIST11 software, following GC/MS analysis was used to determine the natural ratio of the volatiles in the component blend. The volatiles were stored in glass vials at − 80 °C until used for oviposition assays.

#### Oviposition bioassay with identified volatiles

Dual choice bioassay was performed to determine the role of root exudate volatiles in egg laying behavior of *An. gambiae*. Of the volatiles identified, seven compounds: α-pinene, β-pinene, 3-carene, (*E*)-caryophyllene, camphor, α-phellandrene, β-phellandrene were selected and tested based on results obtained from Random Forest Analysis (RFA) (see electronic supplementary material, methods) and their commercial availability^21^. In dose–response assays, several tests were carried out: (i) a blend of the seven components (7-component blend) mimicking their naturally-occurring ratio in the root exudate volatile extract based on their GC/MS peak areas, (ii) single compounds, and (iii) a blend of the attractive components (5-component blend) guided by the positive attractive responses obtained from (ii). The compounds were dissolved in dimethyl sulfoxide (DMSO) and serially diluted from a stock solution to generate a concentration ranging from 0.25 to 4 µg/µL. Thereafter, for each concentration tested, 50 µL of each compound or blend solution were dispensed into the oviposition cup containing distilled (50 mL) water and monitored for egg laying. Each concentration was tested against a control (distilled water (50 mL) containing 50 µL of DMSO). The bioassays were performed in triplicate.

#### Mosquito growth and development assays

The root exudate water and the control water (water from soil) were used separately for mosquito rearing. First, mosquitoes were provided with root exudate water to lay eggs in a no-choice oviposition bioassay. Thereafter, a total of 200 eggs were counted and placed into each rearing tray (24 × 34 × 4 cm) and the number of hatched eggs was determined by counting the first instars larvae that emerged in each tray. The larvae were fed daily on a standardized regimen of ground fish food (Tetramin, Tetra, Germany) and water was changed every other day. Daily survival of larvae (from first instar (L1) to pupation) was recorded, and pupae transferred into cups in experimental cages (30 × 30 × 30 cm) until adult emergence. The conditions of the bioassay room were the same as that of the rearing room described above. All experiments were performed in four replicates.

### Experiments using parthenin

#### Detection of parthenin in root exudate water

One liter (1 L) of water was collected from flooded depressions/open puddles in which wild *P. hysterophorus* was growing on the *icipe* campus and pooled (the field collection was repeated twice, 1 week apart). The habitat had other non *P. hysterophorus* plants such as grasses. The water was filtered using a muslin cloth and stored at − 80 °C overnight and then freeze-dried (VirTis SP scientific, Model Advantage EL-85) for 72 h to obtain 38 mg of root exudate. This was extracted with dichloromethane (DCM) and analyzed by GC/MS as described under chemical analysis. A similar analysis was carried out on the root exudate water (four replicates) obtained from potted plants described above.

#### Preparation of parthenin stock solution

A sample of parthenin obtained from a methanolic extract of *P. hysterophorus* from a previous study conducted in our laboratory^22^, was used as a standard. A sample of this isolate (300 mg) was dissolved in 0.3 mL DMSO and then diluted to 300 mL with distilled water to obtain a stock solution of 1000 ppm.

#### Oviposition response assay with parthenin

Parthenin was evaluated at a concentration of 0.13 µg/µL corresponding to the estimated amount of the root exudate tested (described above). The amount of parthenin in the root exudate was estimated by comparing the relative peak area (%) of parthenin in the root exudate to that recorded for the standard parthenin of known concentration. The oviposition assays were performed as described for root exudate. However, the distilled water which served as a negative control was prepared in 0.1% DMSO. A similar experiment was performed using parthenin water spiked with 50 µL of headspace volatiles collected for 24 h from the plant root exudate. All experiments were performed in triplicates.

#### Mosquito growth and development assays using parthenin

Parthenin prepared as described for the oviposition bioassay was used for mosquito rearing. Mosquitoes reared on distilled water prepared in 0.1% DMSO served as the control group. The experiment was performed as described for root exudate water.

To determine the effect of exposure to parthenin on mosquito adult survival, the female mosquitoes that emerged from parthenin treated water and controls were monitored separately in different cages. The mosquitoes were maintained on *P. hysterophorus* potted plant until their natural death (survival). The conditions in the bioassay rooms were the same as those of the rearing room described above.

### Chemical analysis

#### Gas chromatography/mass spectrometry (GC/MS)

To detect parthenin in the root exudate, the sample was freeze-dried and prepared at a concentration of 600 ng/µL in dichloromethane (DCM) and dried over anhydrous Na_2_SO_4_ (Sigma Aldrich, St Louis, MO USA). The standard parthenin sample was prepared at a concentration of 300 ng/µL in DCM. For GC/MS analysis, 1 µL of each sample (parthenin and root exudate) was analyzed on a 7890B gas chromatograph (Agilent Technologies, Inc., Santa Clara, CA, USA) linked to a 5977A mass selective detector under the following conditions: Inlet temperature 270 °C, transfer line temperature 280 °C, and column oven temperature programmed from 35 to 285 °C, with the initial temperature maintained for 5 min then 10 °C/min to 280 °C for 5.5 min and finally 5 °C/min to 285 °C for 34.9 min. The GC was fitted with a HP-5 MS low bleed capillary column (30 m × 0.25 mm i.d., 0.25 µm) (J&W, Folsom, CA USA). Helium at a flow rate of 1.2 mL/min served as the carrier gas. The mass selective detector was maintained at ion source temperature of 230 °C and a quadrupole temperature of 180 °C. Electron impact (EI) mass spectra were obtained at the acceleration energy of 70 eV. Compounds were injected in the splitless mode using an auto-sampler 7693 (Agilent Technologies, Inc., Beijing, China). Fragment ions were analyzed over 38–550 m*/z* mass range in the full scan mode. The filament delay time was set at 3.0 min. Parthenin was identified based on its general fragmentation pattern and compared also with previously published results^23^. The root exudate samples were analyzed in triplicate, with each replicate collected from a different batch of plants.

### Statistical analysis

The oviposition activity index (OAI) for dual choice oviposition assay data was calculated according to the formula described in^19^;

$$OAI=\frac{\mathrm{Nt}-\mathrm{Nc}}{\mathrm{Nt}+\mathrm{Nc}}$$

where Nt is number of eggs laid in the treatment and Nc the number of eggs laid in the control. The OAI ranges from − 1 to + 1, with 0 indicating neutral response, positive value indicating an attraction towards the treatment and a negative value indicating the converse. The oviposition data was analyzed by generalized linear model using negative binomial. The model validity was assessed by inspection of residuals^24^: Number of eggs deposited served as the response variable while the treatments were used as the predictor variable.

Hatch rate of mosquito eggs exposed to different treatments was calculated as follows:

$$\% hatchability=\left(\frac{number \; of \; hatched \; eggs}{total \;number \; of \; eggs}\right) \times 100$$

The % hatchability data was also analyzed using generalized linear model with negative binomial error structure. The variation in survival was analyzed by the Kaplan-Meir method and statistical significance comparisons made using log-ranks test. All statistical analyses were performed using SPSS 23.0 software (IBM SPSS Statistics) and results considered significance at p < 0.05.

### Ethics statement

Ethical approval for mosquito blood feeding was obtained from Kenya Medical Research Institute (KEMRI) Scientific and Ethics Review Unit (SERU) (Approval number 593). *Plant ethics* Additional approval to obtain samples from the plant was received from National Commission for Science, Technology and Innovation (permit number: NACOSTI /P/20/4177). NACOSTI (http://www.nacosti.go.ke) has a mandate to accredit research institutions and approve all scientific research in Kenya. The current study complied with NACOSTI guidelines (National guidelines).

## Results

### Gravid *An. gambiae* are attracted to root exudate water from wild *P. hysterophorus*

We performed a dual choice oviposition bioassay to test whether gravid *An. gambiae* mosquitoes would lay their eggs in *P. hysterophorus* root exudate water (Fig. 1A). Gravid females were more attracted to lay eggs in the root exudate-treated water, OAI of 0.4, p < 0.001), than in the distilled water control (Fig. 1B,C). However, when presented with a choice between water from soil and distilled water, the oviposition response elicited by the two substrates was not significantly different (OAI of 0.08, p = 0.107, Fig. 1B,C).

### Gravid *An. gambiae* are attracted to volatile terpenes released by *P. hysterophorus* root exudate-treated water

To determine if olfactory cues mediated gravid female attraction to root exudate water, we performed an experiment using volatiles identified from this water sample. First, we performed GC/MS analysis and identified a total of 40 volatile organic compounds comprised mainly of mono- and sesqui-terpenes (electronic supplementary material, Table S1) in the root exudate water. Random Forest Analysis (RFA) identified 16 compounds as the most important (contributing to the attraction of the root exudate-treated water, electronic supplementary material, Figure S1). Of these volatiles, seven components: α-pinene, β-pinene, α-phellandrene, β-phellandrene, camphor, 3-carene and (*E*)-caryophyllene were selected for testing because they were commercially available. Water samples treated with a blend of the seven components, with each component constituted in the natural ratio present in the crude volatiles, attracted gravid females (Table 1). However, when the seven components were tested individually, five components including α-pinene (OAI of 0.37), α-phellandrene (OAI of 0.66), β-phellandrene (OAI of 0.33), 3-carene (OAI of 0.11) and (*E*)-caryophyllene (OAI of 0.76) attracted gravid females, whereas β-pinene (OAI of − 0.28), and camphor (OAI of − 0.09) elicited an avoidance response (Table 2). We then constituted a 5-component blend based on the natural ratios of the individual components that attracted gravid females and compared water treated with this blend to water treated with the crude root exudate volatile extract. The oviposition response elicited by the 5-component blend-treated water was not significantly different from that of the crude volatile extract-treated water (OAI of 0.07, p = 0.144, Table 3). In contrast, the 7-component blend-treated water was significantly less attractive than the crude root exudate volatile extract treated-water (OAI of 0.59, p < 0.001, Table 3). We also compared the oviposition response of the 5-component blend to that of the 7-component blend. Interestingly, the 5-component blend was significantly more attractive than the 7-component blend (OAI of − 0.36, p < 0.001, Table 3). These results confirm the presence of antagonist components in the 7-component blend, which were identified as β-pinene and camphor in tests with the individual components. They also confirm that a blend dominated by specific monoterpenes released by the root exudate attract gravid females.

**Table 1:** Oviposition activity index (OAI) of 7-component blend at different concentrations.

Concentration (µg/µL)	Water Median (range)	7-component Median (range)	OAI	p-value
0.25	168 (168)	239 (132)	0.16	0.002
0.5	108 (119)	98 (200)	0.30	< 0.001
1	93 (75)	219 (115)	0.34	< 0.001
2	151 (82)	145 (111)	− 0.11	0.038
4	41 (360)	68 (30)	− 0.33	< 0.001

**Table 2:** Oviposition activity index (OAI) of individual compounds at optimal attractive dose (1 µg/µL).

Compounds	Water Median (range)	Compound Median (range)	OAI	p-value
α-pinene	24 (9)	0 (141)	0.37	< 0.001
β-pinene	57 (105)	12 (79)	− 0.28	< 0.001
α-phellandrene	10 (56)	66 (154)	0.66	< 0.001
β-phellandrene	37 (47)	66 (154)	0.33	< 0.001
Camphor	4 (95)	40 (47)	− 0.09	0.195
3-carene	21 (55)	38 (57)	0.11	0.148
(*E*)-caryophyllene	3 (17)	39 (39)	0.76	< 0.001

**Table 3:** Binary comparison of oviposition response of blends at optimal attractive doses and crude volatiles.

Median (range)	Median (range)	OAI	p-value
5-component	Crude volatiles		
146 (196)	94 (308)	0.07	0.144

7-component	Crude volatiles		
28 (82)	168 (98)	0.59	< 0.001

5-component	7-component		
105 (153)	102 (114)	− 0.36	< 0.001

### Root exudate water of *P. hysterophorus* influence survival of larval progeny

Since gravid female mosquitoes were attracted to the root exudate volatiles and preferably laid their eggs in root exudate-treated water relative to the control, we tested whether the plant root exudate would influence the bionomics of immature stages of *An. gambiae*. To do this, we compared egg hatch rate, and larval development time to pupation in the root exudate-treated water vs. soil water. The hatch rate in the soil water was 11% higher than that recorded for the root exudate water samples (Fig. 1D). The median survival time to pupation compared favorably to that of the root exudate water (median survival time = 10 days (rep 1), 7 days (rep 2), 9 days (rep 3), no pupation (rep 4)) and the soil water (median survival time = 9 days (rep 1), 7 days (rep 2 & 3), 8 days (rep 4), (*χ*^2^ = 100.0, p < 0.001, Fig. 1E)). These results indicate that some components in the root exudate water reduced egg viability and larval growth and development.

### Gravid *An. gambiae* avoid parthenin-treated water

**Figure 2:**
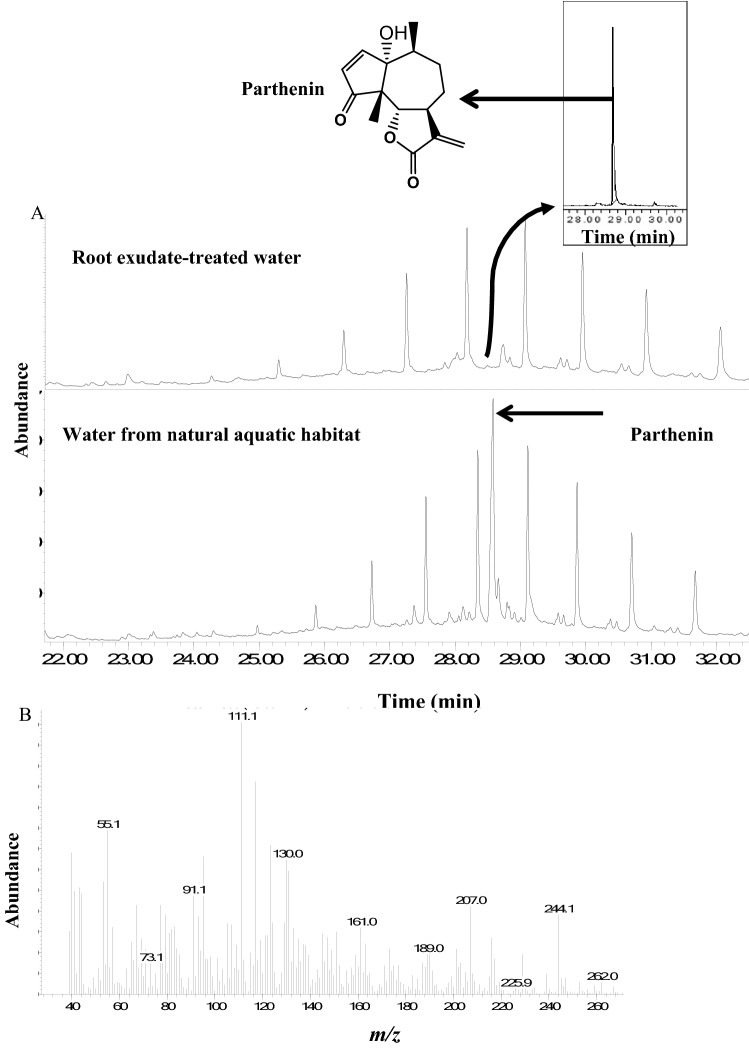
Chemical analysis of root exudate. (**A**) Total ion chromatogram (TIC) showing root exudate profile as detected by GC/MS: Shown in insert is the Extracted ion chromatogram (EIC) of parthenin from the root exudate water sample. (**B**) Mass spectrum of parthenin.

Next, we hypothesized that parthenin, the key secondary metabolite of *P. hysterophorus*, is exuded by the plant roots into water and may influence oviposition behavior and larval growth and development. This hypothesis is based on recent findings that demonstrated that parthenin has larvicidal activity^22^. First, we analyzed the root exudate water samples and natural water collected around *P. hysterophorus* plants in the field by GC/MS and identified parthenin in both samples (Fig. 2A,B). Thereafter, we compared the relative peak area (%) of parthenin in the root exudate to that recorded for the standard parthenin of known concentration, and estimated the release rate of parthenin in the root exudate at 1.2 × 10^–6^ µg/µL/per plant/hr.

**Figure 3:**
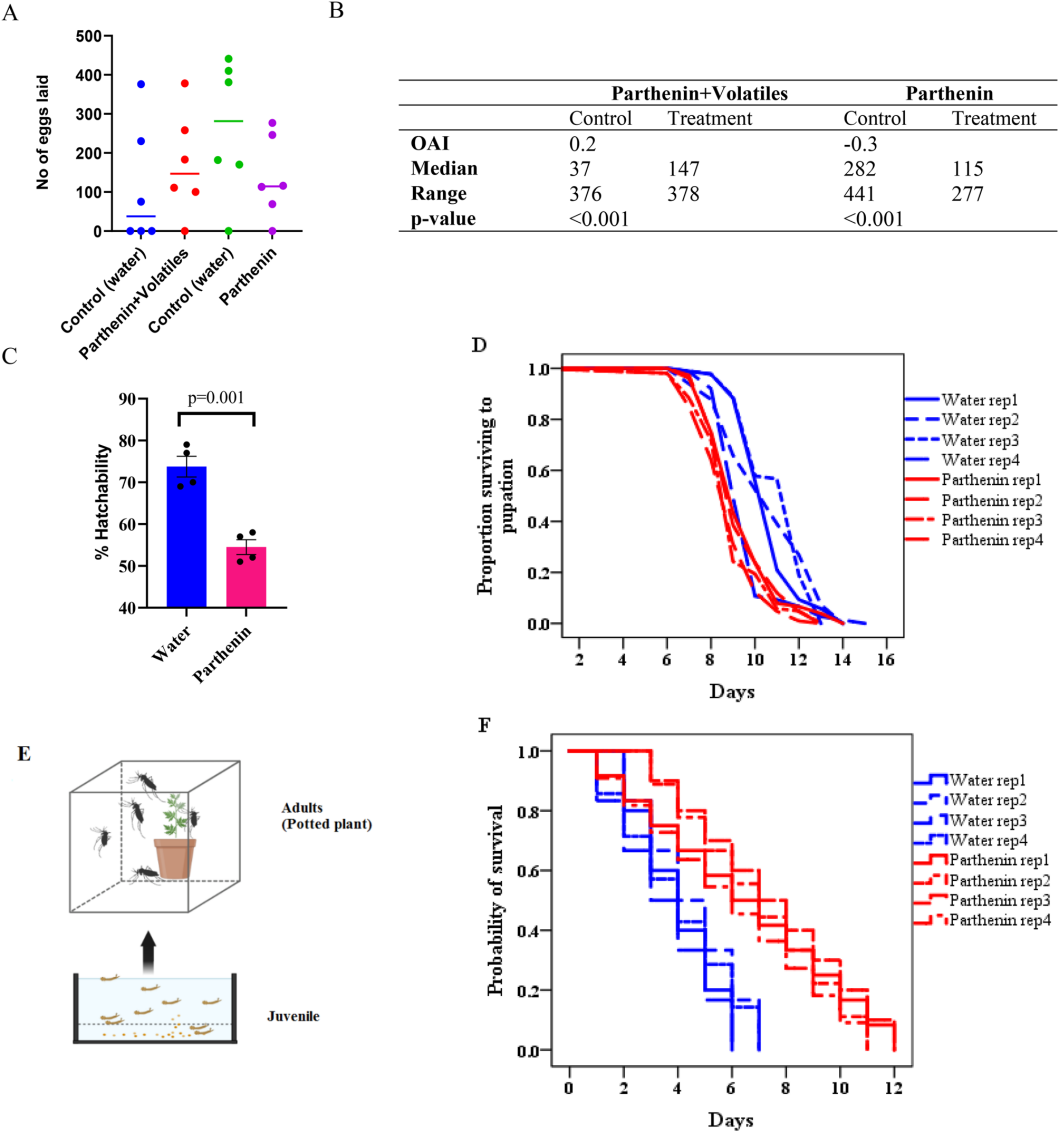
Effect of parthenin on *An. gambiae* oviposition activity and development. (**A**) Oviposition activity of parthenin treated water and distilled (control) water. (**B**) Table summarizing median number of eggs laid and the range. (**C**) Egg hatch rates was low in parthenin water compared to control water. (**D**) Time to pupation (days) for larvae exposed to parthenin relative to control water. (**E**) Pictorial illustration of the survival assays. (**F**) Effect of pre-exposure to parthenin on the survival of *An. gambiae* adult female mosquito. Exposed mosquitoes survived longer than the non-exposed when fed on intact plant.

We tested the effect of parthenin on mosquito oviposition at a concentration of 0.13 µg/µL corresponding to the estimated amount of the root exudate tested under oviposition bioassay. We observed that gravid females avoided parthenin-treated water solutions compared to distilled water (OAI of − 0.3, p < 0.001) (Fig. 3A,B). Additionally, we performed a similar experiment with parthenin-treated water spiked with 50 µL of crude volatiles collected for 24 h from the headspace of root exudate water. Here, mosquitoes were observed to preferably deposit their eggs in the treatment water than in the distilled water (OAI of 0.2, p < 0.001) (Fig. 3A,B). These results further confirm that volatile organic compounds modulate gravid female oviposition behavior.

### Parthenin-exposed eggs increased lifespan in adult females

To determine the effect of parthenin on progeny survival, we monitored the development of eggs laid by gravid females in parthenin-treated solutions and controls. We found parthenin-treated solutions decreased egg hatch rate (Fig. 3C). There was a significant 20% reduction in egg hatch rate in the parthenin-treated water (54% ± 1.8) compared to the control (74% ± 2.5) which indicated that parthenin had mosquito ovicidal activity.

Next, we determined the effect of parthenin on larval growth and survival of adult stages of *An. gambiae* mosquito. The median survival time (in days) to pupation varied between the treatment and the control, with larvae exposed to parthenin developing 2 to 3 days earlier (median survival time = 9 days (rep 1–4)) than the non-exposed control (median survival time = 11 days (rep 1 & 2), 12 days (rep 3), 9 days (rep 4)) (*χ*^2^ = 142.0 df = 7, p < 0.001 Fig. 3D). At the adult stage, female mosquitoes that emerged from parthenin treated water survived 4 to 5 days longer than those that emerged from the control water (*χ*^2^ = 19.3, df = 7, p < 0.007, Fig. 3E,F).

## Discussion

The introduction and spread of invasive plant species that are highly attractive to anopheline mosquito vectors^23^ has called for the need to understand their influence on mosquito behavior, fitness, vectorial capacity, and malaria transmission. In this study, we investigated the influence of *P. hysterophorus* root exudate on *An. gambiae* oviposition behavior and life history traits. Our results confirm that indeed *An. gambiae* females discriminate oviposition sites using olfactory cues^7,9,25^, and demonstrate that the root exudate of *P. hysterophorus* releases a plethora of volatile organic compounds. Some of these components may elicit an attractive or an avoidance response in gravid *An. gambiae* mosquitoes. In the present study, we found that a 7-component terpene blend representing the crude root exudate attracted gravid females to lay their eggs. However, in tests with individual components, two compounds (β-pinene and camphor) elicited an avoidance response. These results suggest that mosquitoes detect a wide range of components, but the decision to locate a host or accept a site as suitable for oviposition is influenced by several factors, including the quality and quantity of the odor signal^4,9,10^. Our results demonstrate that the root exudate of *P. hysterophorus,* dominated by a 5-component blend of terpenes, attracted gravid females. To the best of our knowledge, this is the first time that plant root exudate volatiles have been found to mediate oviposition behavior by gravid *An. gambiae*.

Furthermore, we show that gravid females laid more eggs in the root exudate-treated water than in the water control, which is consistent with previous work on mosquito responses to phytochemicals^25–28^. However, there were potential benefits and costs incurred by females in selecting the root exudate-treated water over water control to lay their eggs. Regarding benefits, it is possible that the gravid females associate the root exudate-treated water with the presence of plant nutrients and microbes for their progeny. On the other hand, by selecting root exudate-treated water to lay eggs, gravid females incurred cost by exposing the juveniles to potential toxic phytochemicals in the root exudate, which resulted in low larval survival. It can also be argued that larval competition for the available nutrients contributed to the low survival rate^6^ however more eggs hatched in the control water than in the root exudate water. Thus, it appears that reproductive investment and survival of progeny in an oviposition site, such as by gravid females in the current study, may be regulated by a range of environmental factors including xenobiotics of phytochemical origin in the aquatic habitat.

Importantly, the selection of oviposition sites with potential xenobiotics of phytochemical origin may provide a trade-off in surviving individuals such as improved fitness. Trade-offs between reproduction, growth and survival is known to influence life history traits and vector competence of various organisms including mosquitoes^26^. For instance, Oliver and Brooke^27^ demonstrated that whereas larvae of *An. arabiensis* tolerated certain heavy metals (cadmium chloride, lead nitrate and copper nitrate), with reduced pupation time, emerged adults were smaller in size but were more tolerant to insecticides. Additionally, it has been shown that the adaptation of anopheline vectors to polluted water can influence adult emergence times^28–30^. Similarly, exposure to sub-lethal doses of insecticides has been reported to increase the life span of infected mosquitoes^31^. In the current study, we found that presence of the sesquiterpene lactone parthenin released from the root exudate of *P. hysterophorus* to the oviposition site environment influenced mosquito bionomics. Although there were costs incurred at the juvenile stages such as reduced egg hatch rates and larval development time to pupal stage, the benefit was evident in adult longevity. Emerged females from parthenin-exposed larvae survived 4 to 5 days longer when fed on the invasive plant, *P. hysterophorus*, compared to their non-exposed counterparts.

The findings from the present study provides a foundation for unraveling how exposure of mosquito juveniles to plant allelochemicals in breeding habitats can influence adult survivorship, which is a critical determinant of vectorial capacity, a metric of transmission potential of an arthropod-borne pathogen^32^. Investigations of this environmental effects on adult survival trait alone is unlikely to provide a clear picture of disease transmission risk and further studies to incorporate other possible interacting factors in the natural setting are warranted. For instance, it would be important to investigate the role of other components identified in the headspace volatiles such as dehydroaromadendrene, zierone and guaiazulene tested both singly and in combination with the 5-component blend in oviposition site selection of gravid females of *An. gambiae*. Future work should also identify other non-volatile components in the root exudate extract and investigate their role in *An. gambiae* female oviposition attraction and bionomics.

## Conclusion

We have demonstrated that the root exudate of *P. hysterophorus* attracts gravid females of *An. gambiae* and envision that root exudates of other plants may also modulate oviposition behavior in other disease vectors. Furthermore, we have shown that exposure to parthenin by the immature stages of malaria vector is associated with increased adult survival. Our findings contribute to the overall knowledge on plant–insect interactions and may inform future studies aimed at developing the next generation of vector control tools.

## Supplementary Information

Supplementary Information.

## Data Availability

The datasets supporting this article have been uploaded as part of the electronic supplementary material.

## References

[1] Sinka ME, Bangs MJ, Manguin S, Coetzee M, Mbogo CM, Hemingway J (2010). The dominant *Anopheles* vectors of human malaria in Africa, Europe and the Middle East: Occurrence data, distribution maps and bionomic précis. Parasit Vectors..

[2] World Health Organization. Fact sheet about Malaria. https://www.who.int/news-room/fact-sheets/detail/malaria, Accessed 7 Jan 2021 (2020).

[3] World Health Organization. The “World malaria report 2019” at a glance. https://www.who.int/news-room/feature-stories/detail/world-malaria-report-2019, Accessed 7 Jan 2021 (2019).

[4] Nyasembe VO, Torto B (2014). Volatile phytochemicals as mosquito semiochemicals. Phytochem. Lett..

[5] Munga S, Minakawa N, Zhou G, Barrack O-OJ, Githeko AK, Yan G (2006). Effects of larval competitors and predators on oviposition site selection of *Anopheles gambiae* sensu stricto. J. Med. Entomol..

[6] Himeidan, Y. E., Temu, E. A., El Rayah, E. A., Munga, S. & Kweka, E. J. Chemical cues for malaria vectors oviposition site selection: Challenges and opportunities. *J. Insects* **2013**, 1–9 (2013).

[7] Lindh JM, Okal MN, Herrera-Varela M, Borg-Karlson A-K, Torto B, Lindsay SW (2015). Discovery of an oviposition attractant for gravid malaria vectors of the *Anopheles gambiae* species complex. Malar J..

[8] Eneh L, Saijo H, Borg-Karlson A-K, Lindh J, Rajarao G (2016). Cedrol, a malaria mosquito oviposition attractant is produced by fungi isolated from rhizomes of the grass *Cyperus rotundus*. Malar J..

[9] Wondwosen B, Birgersson G, Seyoum E, Tekie H, Torto B, Fillinger U (2016). Rice volatiles lure gravid malaria mosquitoes, *Anopheles arabiensis*. Sci. Rep..

[10] Wondwosen B, Birgersson G, Tekie H, Torto B, Ignell R, Hill SR (2018). Sweet attraction: Sugarcane pollen-associated volatiles attract gravid *Anopheles arabiensis*. Malar J..

[11] Kamdem C, Fouet C, Gamez S, White B (2017). Pollutants and insecticides drive local adaptation in African malaria mosquitoes. Mol. Biol. Evol..

[12] Vantaux A, Lefèvre T, Cohuet A, Dabiré KR, Roche B, Roux O (2016). Larval nutritional stress affects vector life history traits and human malaria transmission. Sci. Rep..

[13] Asmare, Y., Hopkins, R. J., Tekie, H., Hill, S. R. & Ignell, R. Grass pollen affects survival and development of larval *Anopheles arabiensis* (Diptera: Culicidae). *J. Insect. Sci.* **17**(5), 1–8 (2017). 10.1093/jisesa/iex067 PMC5597869 28922900

[14] Patel S (2011). Harmful and beneficial aspects of *Parthenium hysterophorus*: An update. 3 Biotech..

[15] CDC-Centers for Disease Control. CDC’s Global Malaria Activities—Kenya. https://www.cdc.gov/malaria/malaria_worldwide/cdc_activities/kenya.html, Accessed 7 Jan 2021 (2019).

[16] Nyasembe VO, Teal PE, Mukabana WR, Tumlinson JH, Torto B (2012). Behavioural response of the malaria vector *Anopheles gambiae* to host plant volatiles and synthetic blends. Parasit. Vectors..

[17] Manda H, Gouagna LC, Nyandat E, Kabiru EW, Jackson RR, Foster WA (2007). Discriminative feeding behaviour of *Anopheles gambiae* s.s. on endemic plants in western Kenya. Med. Vet. Entomol..

[18] Dieter KL, Huestis DL, Lehmann T (2012). The effects of oviposition-site deprivation on *Anopheles gambiae* reproduction. Parasit. Vectors..

[19] Waliwitiya R, Kennedy CJ, Lowenberger CA (2009). Larvicidal and oviposition-altering activity of monoterpenoids, trans-anithole and rosemary oil to the yellow fever mosquito *Aedes aegypti* (Diptera: Culicidae). Pest. Manag. Sci..

[20] Ilahi I, Haq T, Rahim A, Anwar Sajad M, Khan A, Ullah S (2019). Oviposition deterrence and adult emergence inhibition activities of *Cymbopogon nardus *against *Culex quinquefaciatus* with study on non-target organisms. Appl. Ecol. Environ. Res..

[21] Baleba S, Torto B, Masiga D, Weldon C, Getahun M (2019). Egg-laying decisions based on olfactory cues enhance offspring fitness in *Stomoxys calcitrans* L. (Diptera: Muscidae). Sci. Rep..

[22] Milugo TK, Tchouassi DP, Kavishe RA, Dinglasan RR, Torto B (2021). Derivatization increases mosquito larvicidal activity of the sesquiterpene lactone parthenin isolated from the invasive weed *Parthenium hysterophorus*. Pest. Manag. Sci..

[23] Nyasembe V, Cheseto X, Kaplan F, Foster W, Teal P, Tumlinson J (2015). The invasive American weed *Parthenium hysterophorus* can negatively impact malaria control in Africa. PLoS ONE.

[24] Tchouassi DP, Jacob JW, Ogola EO, Sang R, Torto B (2019). Aedes vector-host olfactory interactions in sylvatic and domestic dengue transmission environments. Proc. Biol. Sci..

[25] Melo N, Wolff GH, Costa-da-Silva AL, Arribas R, Triana MF, Gugger M (2020). Geosmin attracts *Aedes aegypti* mosquitoes to oviposition sites. Curr. Biol..

[26] Stearns S (1989). Trade-offs in life-history evolution. Funct. Ecol..

[27] Oliver SV, Brooke BD (2018). The effect of metal pollution on the life history and insecticide resistance phenotype of the major malaria vector *Anopheles arabiensis* (Diptera: Culicidae). PLoS ONE.

[28] Gunathilaka N, Fernando T, Hapugoda M, Wickremasinghe R, Wijeyerathne P, Abeyewickreme W (2013). *Anopheles culicifacies* breeding in polluted water bodies in Trincomalee District of Sri Lanka. Malar J..

[29] Awolola TS, Oduola AO, Obansa JB, Chukwurar NJ, Unyimadu JP (2007). *Anopheles gambiae* s.s. breeding in polluted water bodies in urban Lagos, southwestern Nigeria. J. Vector Borne Dis..

[30] Osse R, Bangana S, Aïkpon R, Kintonou J, Sagbohan H, Aboubakar S (2019). Adaptation of *Anopheles coluzzii* larvae to polluted breeding sites in Cotonou: A strengthening in urban malaria transmission in Benin. Vector Biol. J..

[31] Hauser G, Thiévent K, Koella JC (2020). Consequences of larval competition and exposure to permethrin for the development of the rodent malaria *Plasmodium berghei* in the mosquito *Anopheles gambiae*. Parasit. Vectors..

[32] Tchouassi DP, Torto B, Sang R, Riginos C, Ezenwa VO (2020). Large herbivore loss has complex effects on mosquito ecology and vector-borne disease risk. Transbound Emerg. Dis..

