# Supplementary material for: Root exudate chemical cues of an invasive plant modulate oviposition behavior and survivorship of a malaria mosquito vector
Source: Sci Rep. 2021 Jul 20;11:14785. doi: 10.1038/s41598-021-94043-5 (PMC8292407; doi:10.1038/s41598-021-94043-5)
Supplement: Supplementary file 1 — Supplementary Information. [file 41598_2021_94043_MOESM1_ESM.pdf]

EXPERIMENTAL PROCEDURE

Sample size calculation

Sample size was calculated using the formula for independent samples as described below;

$$N1=\left\{ z1-\alpha/2 * \sqrt{\overline{p} * \overline{q} * \left(1+\frac{1}{k}\right)} + z1-\beta * \sqrt{p1 * q1 + \left(\frac{p2 * q2}{k}\right)} \right\}^{2}/\Delta 2$$

q1=1−p

q2=1−p2

$\overline{p}=\frac{p1+kp2}{1+k}$

$\overline{q}=1-\overline{p}$

$$N1=\left\{ 1.96\sqrt{0.5 * 0.5 * \left(1+\frac{1}{1}\right)} + 1.28 * \sqrt{0.9 * 0.1 + \left(\frac{0.1 * 0.9}{1}\right)} \right\}^{2}/0.8^{2}$$

N1=6

N2= K∗N1=6

Therefore, total sample size = N1+N2=6+6=12

Where
N1 and N2 is the sample size for group 1 and 2 respectively, p1 and p2 is the estimated proportion of the two groups, Δ is the absolute difference between the two proportions, α = 0.05 and is the probability of type I error while β = 0.1 and is the probability of type II error, z is the selected critical value of desired confidence level in this case 95% and K is the ratio of sample size for the two groups

Random Forest Analysis (RFA) calculation

For Random Forest Analysis (RFA), the random forest package in R software version 3.4.3 (R core development team) was used to identify a minimum set of variables (VOCs in this case) that are abundant and permanently present (most important volatile) in root exudate water obtained from plants at different growth stages (i.e., non-flowering and flowering; electronic supplementary material, methods). The relative abundance of each identified volatile organic compound following GC/MS analysis was used in the RFA. The RFA was executed by running 100,000 iterations (ntree) with 6 volatiles randomly selected at each split ($mtry=\sqrt{q}$ where q is the total number of volatiles) from a total of 40 variables (VOCs). The function 'importance' was used to generate the mean decrease in accuracy (MDA) and volatile with the highest value considered the most important (20).

Plants: Seeds were collected from wild *P. hysterophorus* plants growing at the International Centre of Insect Physiology and Ecology (*icipe*) garden, Duduville campus, Nairobi (1°16′60″S; 36°49′0″E) in June, 2019, three weeks after the onset of the rainy season. The seedlings were air-dried and planted in plastic pots (23 cm top diameter, 12 cm base diameter and 22 cm depth) with sterilized sand (autoclaved at 121 °C for 40 minutes) and grown using nutrient solution as previously described. The plants were watered daily in the morning and maintained in a screenhouse at 22 ± 2 °C and 60–70% relative humidity (RH). Once at the flowering stage, the plants were removed from the plastic pots gently, avoiding root damage, and the roots wrapped immediately with aluminum foil to maintain the below ground environment. The plants were transported thereafter to the laboratory and roots washed gently with distilled water to remove soil. Batches of ten plants were soaked in 500 ml of distilled water in a glass beaker for 24 h at room temperature. The plants were removed and discarded and the water containing the exudate filtered using a muslin cloth. The filtrate was stored at -80 ^{o}C overnight and then freeze-dried (VirTis SP scientific, Model Advantage EL-85) for 72 h to obtain the root exudate (32 mg). This experiment was performed on plants at different growth stages (pre- and post- flowering) to obtain root exudate water for chemical analysis.

Table S1: Compounds detected in root exudate volatiles

RT_{min}	Compound name	Class of compound
9.84	α-Pinene	Monoterpene
10.17	Camphene	Monoterpene
10.76	β-Pinene	Monoterpene
11.32	α-Phellandrene	Monoterpene
11.56	2-Carene	Monoterpene
11.79	β-Phellandrene	Monoterpene
12.96	3-Carene	Monoterpene
13.81	Camphor	Monoterpene
14.17	Borneol	Monoterpene
15.00	γ-Terpinene	Monoterpene
15.97	Cogeijerene	Sesquiterpene
16.65	δ-Elemene	Sesquiterpene
17.14	α-Ylangene	Sesquiterpene
17.23	Dauca-5,8-diene	Sesquiterpene
17.40	β-Elemene	Sesquiterpene
17.56	β-Guaiene	Sesquiterpene
17.81	E-Caryophyllene	Sesquiterpene
17.90	Bicyclogermacrene	Sesquiterpene
17.96	α-Guaiene	Sesquiterpene
18.03	6,9-Guaiadiene	Sesquiterpene
18.09	Zonarene	Sesquiterpene
18.41	E -Calamenene	Sesquiterpene
18.45	α-Humulene	Sesquiterpene
18.55	γ-Himachalene	Sesquiterpene
18.61	α-Gurjunene	Sesquiterpene
18.67	δ-Selinene	Sesquiterpene
18.71	γ-Gurjunene	Sesquiterpene
18.98	Epishyobunone	Sesquiterpene
19.28	z-Calamenene	Sesquiterpene
19.36	Selina-3,7(11)-diene	Sesquiterpene
19.56	β-Calacorene	Sesquiterpene
19.90	γ-Muurolene	Sesquiterpene
20.22	E-Cadina-1(6),4-diene	Sesquiterpene
20.32	β-Vetivenene	Sesquiterpene
20.70	Selin-11-en-4-alpha-ol	Sesquiterpene
20.84	α-Himachalene	Sesquiterpene
20.95	Zierone	Sesquiterpene
21.98	Guaiazulene	Sesquiterpene
23.74	dehydro-Aromadendrene	Sesquiterpene

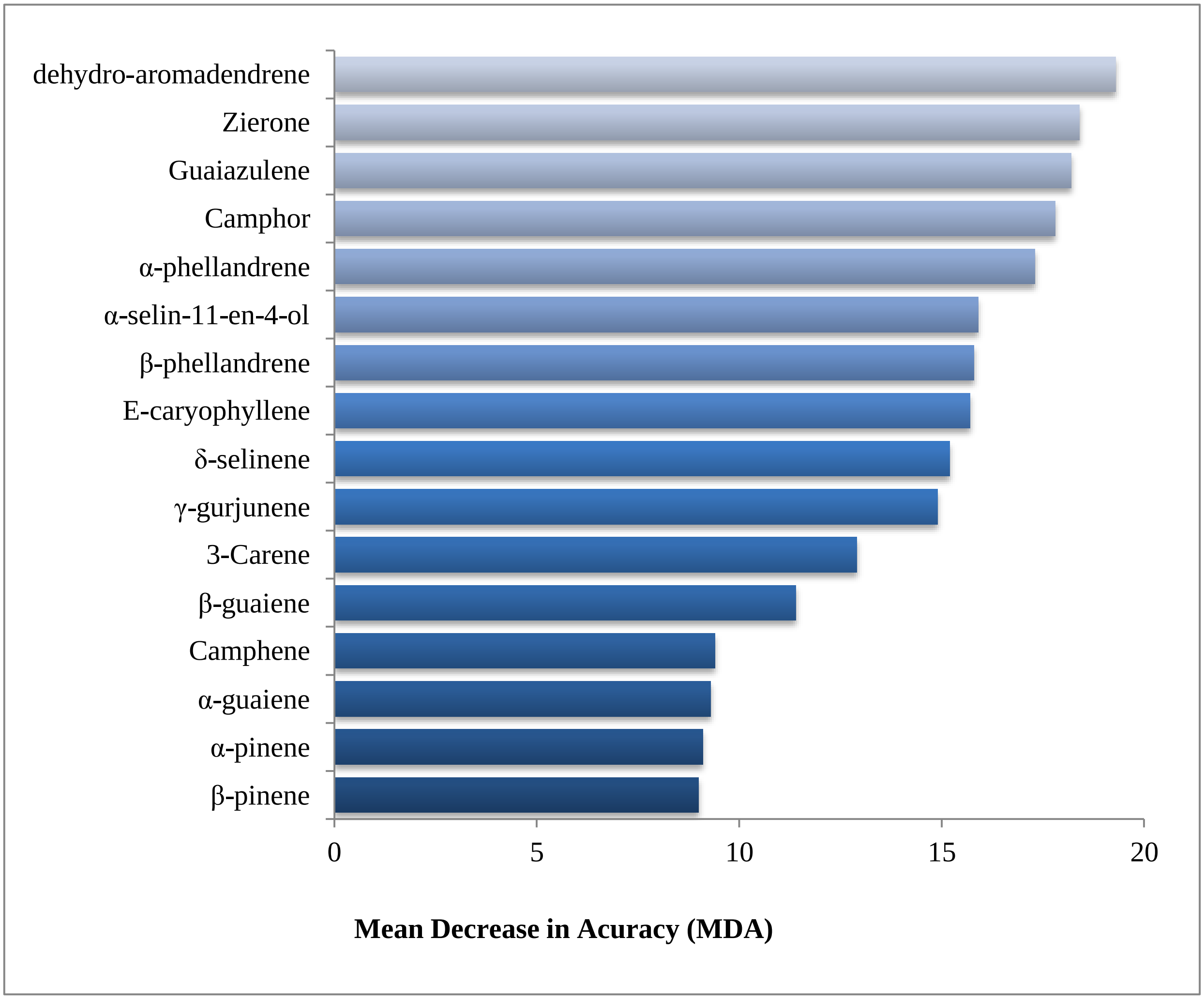

Figure S1: Random Forest Analysis (RFA) results showing compounds importance based on their Mean Decrease in Accuracy values. Volatile organic compounds (VOCs) associated with light blue histograms have the highest MDA and consequently the most important.
